# Autograft Enlargement After the Ross Procedure in Pediatric Patients: Somatic Growth or Pathologic Dilatation?

**DOI:** 10.1177/21501351251400224

**Published:** 2026-01-14

**Authors:** Ioannis Zoupas, Alexander C. Mills, Scott D. Olson, Damien J. LaPar

**Affiliations:** 1 Department of Pediatric Cardiovascular Surgery, 12340 Children's Heart Institute, Children's Memorial Hermann Hospital, 12340 The University of Texas Health Science Center at Houston, Houston, TX, USA; 2 Department of Pediatric Surgery, 12340 The University of Texas Health Science Center at Houston, Houston, TX, USA

**Keywords:** aortic valve replacement, Ross, pediatric, autograft, congenital

## Abstract

**Purpose of Review:**

Pulmonary autograft autotransplantation represents a popular surgical approach for pediatric patients requiring aortic valve replacement due to the potential for autograft enlargement to accommodate somatic growth. Nevertheless, autograft dilatation and the subsequent need for reintervention are quite common. Published data suggest that autograft enlargement may result from pathological passive remodeling rather than active somatic growth and vice versa. The present review serves to comprehensively evaluate available evidence related to the fate of the pulmonary autograft after the Ross procedure as it relates to the etiology, risk factors and patterns of autograft failure.

**Results:**

Studies present conflicting results supporting both pathological dilation and active somatic growth. Primary factors impacting the successful remodeling of the autograft include native aortic and pulmonary valve anatomy, medical history, concomitant procedures, perioperative management, age at the time of surgery, and the surgical technique used for the Ross procedure.

**Conclusion:**

Autograft enlargement after the pediatric Ross operation may result from either somatic growth or passive dilation or a combination of both factors. Distinguishing between the two primary etiologies of autograft enlargement depends upon meticulous patient selection and a surgical strategy precisely tailored to individual anatomy and risk.

## Introduction

Pediatric aortic valve replacement (AVR) remains a major surgical challenge due to the limitations of available prosthetic options. Mechanical valves require lifelong anticoagulation and are associated with thromboembolic and hemorrhagic complications, while bioprosthetic and homograft valves often undergo rapid degeneration and failure in the pediatric population.^1–5^ Additionally, limitations surrounding the availability of appropriately sized prostheses or valves as well as the inability of nonliving valves to accommodate somatic growth often necessitates multiple reoperations during childhood. The Ross procedure, autotransplantation of the pulmonary valve into the aortic position, addresses many of these limitations by providing a living, autologous valve substitute capable of growth, adaptive remodeling, and native-like hemodynamics.^1–3,6,7^ The Ross autograft eliminates the need for anticoagulation and is thought to allow the neoaortic autograft to grow with the child.^6,8–10^

Despite its advantages, the Ross procedure is not without limitations. Autograft dilatation and progressive aortic insufficiency (AI) remain significant challenges, particularly when the dilation is disproportionate to somatic growth, potentially requiring reoperation.^7,11–14^ While the pulmonary autograft is ideal due to its growth potential,^9,15^ adverse remodeling and progressive dilatation may lead to severe complications such as neoaortic regurgitation, dissection or sinus rupture.^16,17^ No current surgical technique for the Ross autograft reliably prevents such outcomes while preserving growth potential,^17^ and detailed histologic studies of well-functioning autografts are still lacking. Thus, the biological mechanisms of adaptation remain poorly understood.^18^ Moreover, while excellent outcomes have been reported in children, the neonatal Ross procedure is associated with high early mortality, and treatment of unreparable aortic valves in neonates remains an unresolved problem in congenital cardiac surgery.^19^ Although the Ross procedure offers survival and hemodynamic advantages over mechanical and bioprosthetic AVR in pediatric patients,^20–26^ its success and freedom from autograft reoperation are deemed to be subject to careful timing and patient selection.^27^

Whether the pulmonary autograft enlargement that occurs during follow-up is attributed to active somatic growth and/or passive dilatation remains a topic of debate. This review aims to comprehensively evaluate all available evidence related to the fate of the pulmonary autograft after the Ross procedure with particular attention to biomechanical characteristics, timeline, patterns, and risk factors for dilatation.

### Autograft Autotransplantation in the Pediatric Population

Several studies have supported the concept that the autograft size increase closely reflects increases in body surface area, suggesting active somatic growth rather than pathological expansion.^11,28,29^ Data from multiple pediatric cohorts and meta-analyses show that the pulmonary autograft can increase in diameter concomitantly with somatic growth, particularly at the annulus, but that the neosinus and sinotubular junction (STJ) may undergo more pronounced or disproportionate dilation over time.^11,28–30^ Nevertheless, reports of autograft dilation without significant regurgitation^31,32^ and variable z-score progression at the sinus and STJ^29^ highlight the heterogeneity of Ross outcomes in pediatric patients, in terms of success rates, failure patterns, and risk factors.

Survival and freedom from autograft reintervention in pediatric Ross cohorts have been reported across a broad but encouraging range. Center-specific cohorts have reported freedom from autograft reoperation ranging from 74% to 98% at 10 years, depending on age, follow-up length, and surgical era.^7,9,29,33–38^ In a single-center cohort of 240 children, Nelson et al reported a 15-year freedom from autograft reintervention of 59%,^33^ while another cohort reported 94.1% and 74.9% freedom at 20 years for children and adolescents, respectively.^9^ Schneider et al documented a cumulative incidence of reoperation of 31% at 20 years in a mixed-age population.^34^ In a pooled meta-analysis of 18 studies, the estimated autograft reintervention rate was 19.2% (95%: 7.3%-34.5%), highlighting the variability across centers and patient populations.^32^ Neoaortic root dilatation with moderate to severe AI was the most common indication for autograft reoperation, accounting for approximately half of the reinterventions in some series.^9,39^ Given this, more recent data suggest that with selective application of the Ross procedure, particularly in non-neonatal populations and with refined techniques, autograft failure rates may be substantially reduced, with some studies reporting freedom from autograft failure exceeding 90% at 5 years and over 80% at 15 years in selected patients.^7,40,41^ According to existing literature, autograft failure is influenced by age at surgery, preoperative diagnosis and anatomy, usage of specific surgical techniques, and rapidness of somatic growth during early follow-up.^1,5,7,9,29,31,42–44^

### Preoperative Diagnosis, Anatomy, and Medical History Characteristics

Among the most critical elements affecting autograft success in pediatric patients is the preoperative diagnosis, with primary AI consistently associated with reduced autograft durability and higher rates of dilation and reoperation compared with aortic stenosis (AS).^28,44–46^ Patients with AI may harbor underlying genetic vasculopathies, such as connective tissue disorders, that may impair the autograft's adaptive remodeling capability, contributing to early failure.^47^ Additionally, a preoperatively dilated native aortic annulus (≥27 mm or indexed >16 mm/m²) and aortic to pulmonary annular diameter ratio greater than 30% or diameter mismatch greater than 2 mm are predictive of early autograft dilation, likely due to mechanical overstress and prestretch of the autograft at implantation.^1,18,44,48–50^ This mismatch is particularly problematic in neonates and infants, who often present with congenital AS and a small aortic annulus juxtaposed with a relatively large pulmonary root.^5,40,44^ In particular, a native pulmonary valve diameter >24 mm has been identified as a threshold above which the risk of autograft reoperation increases significantly.^9^ In terms of bicuspid aortic valve (BAV) aortopathy, while a BAV itself is not a direct risk factor for autograft failure, all reported cases of autograft dissection occurred in BAV patients, highlighting the need to understand underlying mechanobiological vulnerability.^16,18^

Underplaying patient-related factors have the potential to impact the natural history of an implanted autograft. Factors such as male sex and immediate postoperative hypertension have been associated with higher autograft dilatation risk.^18,43,44^ Surgical history also plays a significant role, including prior ascending aortic replacement and the need for a Konno annular enlargement are associated with increased autograft stress and worse outcomes.^7,20^ Lastly, Marfan syndrome and related aortopathies represent clear contraindications for the Ross operation due to structural abnormalities of the pulmonary artery wall, which predispose to aneurysm and rapid dilation disproportional to somatic growth.^51,52^ Together, these findings underscore the importance of comprehensive preoperative assessment—integrating diagnosis, annular dimensions, anatomic compatibility, and genetic predisposition—to optimize patient selection and minimize autograft-related complications.^40^

### Age-Dependent Outcomes of Autograft Durability

The performance and long-term durability of the pulmonary autograft in pediatric patients vary significantly across age groups, with important distinctions seen between infants, younger children, and adolescents. Accumulated data have reported that neonates and infants generally experience better early autograft adaptation and durability, potentially due to intrinsic histologic advantages of the pulmonary root at younger ages, including higher proportions of immature cells, elastin, collagen, a closer embryologic similarity to the aortic root and the absence of the remodeling process that occurs after long exposure to the systemic circulation.^14,53,54^ As a result, some data suggest that infants may have lower rates of autograft reintervention than older children, and their autografts expand proportionally with somatic growth rather than undergoing pathologic dilation.^15,43,55,56^ This result may also be related to the neonatal pulmonary valve's prior exposure to high pulmonary vascular resistance during embryonal life, which could render it more resilient to systemic pressures.^5,7^ Conversely, adolescents appear to have higher risks of autograft dilation and reoperation, with adolescent age at surgery being a significant predictor of autograft failure. In one study, freedom from autograft reoperation was 94.1% in young children versus 74.9% in adolescents at 20 years of follow-up, and adolescence itself was identified as an independent risk factor (hazard ratio 3.9, *P* = .032).^9^ One explanation for these observations may relate to the observations that neoaortic annular and sinus z-scores tend to stabilize in young children postoperatively but increase significantly in adolescents, with dilation especially pronounced during the first postoperative year.^29,43,44^

However, despite the advantages of infant Ross compared with Ross in older children in the aforementioned studies, an equal representation of data suggesting a disadvantage of infant Ross exists as well. Infant and neonatal autograft durability are not uniformly superior, and several studies report lower freedom from reoperation in neonates and infants compared with older children. In a pediatric Ross cohort by Donald et al, the freedom from autograft reoperation rate was 61.6% in neonates and infants versus 82.5% in older children at 15 years. Furthermore, this report demonstrated that age <1 year at the time of surgery increased the likelihood of developing moderate or severe postoperative AI.^42^ Other cohorts and meta-analyses reflected this variability, reporting autograft reintervention rates between 0% and 7.5% in neonates/infants, with no consistent benefit from early “autograft training” through neonatal pulmonary pressure exposure.^6,32^ In contrast, children 18 months to 8 years of age may represent a transitional group with stable annular dimensions and minimal—disproportional to somatic growth—sinus z-score progression, potentially offering the best balance between remodeling capacity and durability.^44^

Based upon the evaluation of accumulated data examining the impact of pediatric age on the fate of the Ross autograft over time, surgical strategies tailored to neonates, infants, older children, and adolescents seem to be essential to improving long-term outcomes across the pediatric age spectrum. Current evidence suggests that delaying the Ross procedure until after infancy is beneficial, and that valve repair should be pursued as long as possible whenever feasible.^9,42,43^

### Section-Specific and Timing-Related Autograft Dilatation Patterns

A considerable body of evidence suggests that the most critical period for autograft dilation occurs within the first postoperative year, with up to 60% of total dilation occurring before hospital discharge and a sharp increase in annular and sinus z-scores noted by 12 months.^5,28,31^ According to Williams et al, one of the largest published pediatric Ross experiences, the annulus and sinus experience the most significant expansion, with mean z-scores rising from baseline to 3.2 and 2.5, respectively.^5^ This rapid, passive dilation has been attributed to abrupt exposure of the pulmonary root to systemic pressure, particularly affecting the sinus and STJ, the latter being especially prone to dilatation due to its role in aortic expansion during systole.^28,57^ Several studies report that sinus dilation outpaces annular growth early on, and that the STJ is a common site of structural compromise leading to progressive AI.^28,31,42^ Although early z-score increases are significant, many groups describe subsequent stabilization of the autograft dimensions beyond the first postoperative year, especially in younger patients, suggesting a transition from passive dilation to active, somatic, physiological growth. This actively supports the argument that the autograft should be “protected” through aggressive medical management of systemic blood pressure to avoid hypertension throughout the initial postoperative period.^5,28,44,58^

Longitudinal data suggest that autograft failure due to dilation is predominantly a late event, with reoperations often occurring more than five years after autograft implantation.^13,27,39,59–61^ While patients undergoing the Ross operation in infancy demonstrate early sinus dilation, this trend appears to plateau over 8 to 10 years, representing normal sizing potentially due to better adaptive remodeling.^44,62^ Conversely, patients operated on in later childhood or adolescence experience more prolonged and pathological sinus dilation, with a steeper dilation curve within the first five years post-op.^44^ Eventually, in terms of neoaortic root sections and somatic growth, the annulus tends to grow proportionally with somatic growth, while the sinus and STJ continue to dilate beyond physiological expectations, pointing toward passive mechanical stress as the underlying mechanism.^28,30,31^ The differential remodeling of various autograft sections emphasizes the importance of early hemodynamic control and targeted reinforcement, especially of the STJ, to reduce long-term complications. Ultimately, while the annular z-score increases often parallel body growth, sinus and STJ dilation patterns are less predictable and more closely associated with autograft failure.^28,31,42,57^

### Impact of Surgical Techniques in Autograft Success Rates

Various surgical implantation techniques have been described for the pulmonary autograft during the Ross procedure. Among described techniques, the freestanding full-root technique, while anatomically versatile and more broadly applicable across varying valve anatomies, is consistently associated with increased rates of autograft dilatation and subsequent AI, particularly in children and adolescents.^18,39,42^ Studies have shown that dilatation occurs almost exclusively following the root technique, with nonstructural degeneration, defined by wall dilatation >50 mm and subsequent AI, being the primary mechanisms of failure.^63^ On the other hand, according to Elkins et al and other groups, the intra-aortic subcoronary technique is associated with superior long-term durability, reduced dilatation, and preservation of valve architecture, despite its technical complexity and risk of distorted leaflet.^63–67^ Postexplant analyses confirm that leaflets implanted via subcoronary methods maintain better structural integrity than those implanted through full-root replacements.^53,66,68,69^

In pediatric patients, reinforcement strategies such as neoaortic annular or STJ support have shown benefit in limiting disproportionate dilatation,^20,42,70^ yet are used cautiously in neonates, infants, and younger children so as not to interfere with somatic growth potential.^32^ On the other hand, for the adolescent population, where the rate of somatic growth may be less dramatic, root reinforcement using prosthetic or autologous materials, while potentially protective, must take into account that careful balance between overrestriction that may inhibit remodeling and induce atrophy as well as insufficient support that may allow for pathologic dilation.^18,71^ Additional technical factors such as high autograft implantation (supra-annular positioning), degree of annular support, and technique-specific stress distributions all contribute to varying autograft success rates.^31,32,44^ Van Hoof et al also suggested that bringing the autograft suture line deeper into the left ventricular outflow tract and benefiting from native fibrous support created by suture lines has been associated with less dilation.^18^ Overall, a reliable reinforcement technique that actively prevents dilation, while also allowing somatic growth has yet to be discovered.

### Mechanobiological Properties of the Dilated and Nondilated Autograft

Postimplantation, the mechanobiological behavior of the pulmonary autograft is the result of complex interactions between biomechanical stress and cellular adaptation. Several studies underscore the embryological relation between the aortic and pulmonary roots, suggesting the potential of the autograft to remodel into an aortic phenotype.^48,66,72^ However, the abrupt hemodynamic shift from low-pressure pulmonary to high-pressure systemic circulation triggers intense mechanical strain on a vessel structurally ill-equipped for such an environment.^48^ The pulmonary root lacks the fibrous annulus and ventricular support of the aortic root, originating instead from the thin-walled, muscular right ventricular outflow tract.^73^ As a result, the autograft is immediately subjected to relative supraphysiological distension, most acutely at the sinus and STJ, whereby higher z-scores are observed postimplantation.^20^ This distension induces a cell-mediated remodeling response through mechanotransduction pathways, with smooth muscle cells (SMC) and fibroblasts sensing stress through extracellular matrix (ECM)-integrin interactions and initiating production of structural proteins, matrix metalloproteinases (MMPs), and adhesion molecules.^74^ The anatomical limitations of the pulmonary autograft, namely, its thinner wall, fewer elastic layers, and higher compliance, may predispose it to maladaptive changes during remodeling, rather than sustained physiologic adaptation.^28,74,75^

Maladaptive remodeling in failed autografts is characterized by a specific proteomic and mechanical signature distinct from normal pulmonary arteries or successfully functioning autografts. Chiarini et al^76^ demonstrated that dilated Ross autografts exhibit upregulation of vimentin (VIM) and paxillin, markers of dedifferentiated and artificial SMC phenotypes, as well as soluble Jagged-1 fragments and ectodysplasin-2 receptor, coupled with decreased levels of MAGP-1 and Notch1. This proteomic profile suggests destabilization of the cytoskeleton and impaired elastic fiber assembly. Specifically, the reduction in MAGP-1 implies compromised integrity of the elastic lamellae, while upregulated VIM points to a vascular injury–associated SMC phenotype. These proteomic alterations are not limited to grossly dilated neoaortic roots as even moderately dilated autografts reveal significant dysregulation.^76^ As a result, mechanically, failed autografts demonstrate increased compliance and decreased stiffness when tested ex vivo, compared with both native pulmonary autograft and aortic roots under equivalent pressures.^77^ Notably, wall stiffness did not correlate with patient age or duration in systemic circulation, reinforcing that the failure mechanism is not merely a function of time or growth, but of intrinsic biologic processes in response to pressure. Histologically, these failed roots exhibit elastic fiber fragmentation, adventitial fibrosis, increased collagen deposition, and wall thickening, findings that differentiate them from autografts with stable dimensions.^53,69,77,78^

While viable endothelial and interstitial cells are present in both successful and failed explants,^79^ only the latter show features such as reduced stiffness, persistent ECM turnover, and pronounced adventitial collagen accumulation after 10+ years postimplantation.^53,76,80^ This ongoing remodeling, with elevated MMP1, MMP13, TGF-β, and myofibroblast activity, indicates a chronic maladaptive fibrotic response rather than homeostatic repair, and the eventual inability to restore a normal homeostatic state.^53,69,80^ Even well-functioning autografts may exhibit thickened valve leaflets and pannus formation, signs of adaptation to stress that may portend future dysfunction.^53,66,69^ In rare cases, long-term functioning autografts show organized elastic lamellae and improved microarchitecture, suggesting partial remodeling potential.^66^ However, most evidence points toward a scenario in which early passive distension,^28,31^ uncorrected stress burden,^81^ and progressive ECM degradation result in irreversible pathological dilation.

This understanding reinforces the need for aggressive blood pressure control, especially in the early postoperative period, to ensure a smooth transition of the autograft to the systemic circulation and kickstart beneficial cellular remodeling,^82^ reinforcement of the autograft wall, when feasible, and cautious patient selection for the Ross procedure in growing children.^40^

### Animal Studies on Autograft Remodeling and Functionality

Animal studies represent a fundamental part in Ross autograft research because they allow the investigation of healthy, nondilated pulmonary autografts after explantation, an opportunity unavailable in human patients, where tissue is typically retrieved only after failure. These models provide critical insights into the remodeling capacity of the autograft under systemic load. In ovine and porcine models of the Ross operation, explanted autografts demonstrated preservation of the trilayered valve leaflet architecture and normal cellular distribution up to 22 ± 2.7 months postimplantation,^83^ along with neovascularization at the leaflet base and native cell populations in both valve and wall.^78,84^ Moreover, even with transection of the vasa vasorum, normal vessel growth was preserved in isolated segments,^85^ countering the hypothesis that vasa vasorum transection impairs remodeling. The vessel wall exhibited revascularization and partial restoration of vasa vasorum in both animal and human studies, suggesting that concerns about harvesting-related ischemia may be mitigated by intrinsic vascular ingrowth.^66,78,80^

Remodeling features such as SMC hypertrophy, increased collagen content, and preserved wall microarchitecture were frequently observed, while valve enlargement exceeded what would be expected from somatic growth alone, indicating a biologically active response to mechanical load.^84^ Biaxial mechanical testing in interposition graft models revealed that some pulmonary arteries developed aorta-like mechanical behavior, while others showed vascular atrophy, highlighting heterogeneity in the remodeling response.^86,87^

External resorbable support was shown to prevent excessive dilation and allow growth-compatible expansion of the autograft, although overly rigid materials led to wall erosion and local atrophy.^88,89^ Notably, in Lewis rats with heterotopically implanted syngeneic pulmonary autografts, early increases in diameter and subsequent stiffening were observed via serial ultrasound and mechanical testing. Histologic analysis at two months revealed intimal hyperplasia and focal thinning of elastic lamellae, likely contributing to the increased wall rigidity.^90^ However, it remains uncertain whether new elastin produced in adult animals meaningfully restores mechanical resilience, and the young age and short follow-up in animal models limit direct applicability to the decades-long progression of human autograft failure.^74^ Nonetheless, these findings affirm that the pulmonary autograft possesses an intrinsic, although heterogenous and limited, ability to remodel under systemic stress and that animal models remain indispensable for dissecting the early mechanobiological responses of the nonfailed autograft.

## Comments

Accumulated data and general consensus suggest that the Ross procedure provides an advantageous and suitable intervention for pediatric patients with congenital aortic valve pathologies^40,91^ that require AVR when valve repair techniques and valve-sparing procedures may not be suitable,^29,91^ as prior root operations are not considered a deterrent for a Ross.^92^ Moreover, many series identify a possible volume-outcome relationship when it comes to select, higher-risk patient populations such as neonates and infants.^40^

With respect to the fate of the pulmonary autograft and potential for pathophysiologic enlargement, neoaortic root dilatation and subsequent regurgitation are common but do not frequently occur before adulthood.^7^ Whether this enlargement results principally from active somatic growth or pathologic dilatation, conflicting evidence exists to support each theory. Certain data suggest that the aortic and pulmonary root, especially in older children and adolescents, have drifted far apart from their common embryonal origin; hence, making the autograft unable to truly achieve mechanical homeostasis, comparable to that of a native aorta.^18^ This potential may differ in neonates and infants, where such divergence has yet to occur. Evidence has also revealed that the neoaortic annulus diameters tend to normalize in younger children and that autograft dilatation and regurgitation primarily affect older pediatric populations, in whom root supportive measures may be necessary.^30,43^ Histologic studies have demonstrated normal tissue viability and cellular architecture in pulmonary valves explanted from the aortic position, suggesting active somatic growth.^93^ Nevertheless, this is believed to occur concomitantly, at least partially, with passive dilatation, further contributing to the heterogeneity of existing evidence.^94^

Multiple studies have demonstrated that the Ross procedure in pediatric patients yields significantly superior results compared with mechanical, allograft, or bioprosthetic valve replacements. Large retrospective analyses and meta-analytic studies further indicate lower early and late mortality following the Ross procedure relative to both mechanical and homograft valves.^91,95–99^ Microsimulation studies and national registry analyses have confirmed that the Ross procedure in children and young adults achieves survival rates approaching those of the age-matched general population, an outcome not replicated with mechanical or bioprosthetic valve replacements.^96,97,99^ In addition, the Ross procedure is associated with significantly lower rates of thromboembolic events, major bleeding, and endocarditis compared with both mechanical and bioprosthetic valve replacements.^95,99,100^ The most plausible explanation is the hemodynamic superiority of the Ross procedure, characterized by lower residual transvalvular gradients and superior valve function compared with mechanical and bioprosthetic prostheses.^42,95^

Lastly, in recent years, the application of fresh aortic transplantation for complex aortic valve/root congenital heart disease has also gained increasing attention. Reported early to mid-term results of this technique have demonstrated its surgical application and reproducibility for severe truncal valve and aortic valve disease.^101^ While the mid- to long-term results of this approach and the fate of the graft have yet to be established, this technique may reveal itself to be an attractive surgical option for select patients in the future.

## Conclusion

For pediatric patients and young adults in whom all options for aortic valve repair have been exhausted, the Ross procedure remains the preferred and most physiologically appropriate option for AVR. Postprocedural autograft enlargement represents a complex and multifactorial phenomenon, and ongoing debate persists as to whether it reflects true somatic growth or pathological passive dilation. While the pulmonary autograft demonstrates growth potential, particularly at the annulus, the more disproportionate expansion of the sinus of Valsalva and STJ often suggests passive, pathophysiologic dilation. Factors such as preoperative AI, annular mismatch, concomitant procedures, and connective tissue disorders are key contributors to adverse remodeling and disproportionate dilation. On the other hand, the choice of the correct surgical technique for the corresponding age and modifications such as external annular support and/or reduction, sinus and STJ external support and aggressive management of postoperative systemic hypertension all significantly improve long-term durability. Ultimately, individualized surgical planning remains essential in optimizing outcomes and maximizing autograft longevity.

## Abbreviations

AI: aortic insufficiency
AS: aortic stenosis
AVR: aortic valve replacement
BAV: bicuspid aortic valve
MMP: matrix metalloproteinase
STJ: sinotubular junction
VIM: vimentin
